# VGLUT2/Cdk5/p25 Signaling Pathway Contributed to Inflammatory Pain by Complete Freund's Adjuvant

**DOI:** 10.1155/2020/4807674

**Published:** 2020-02-29

**Authors:** Yuwen Tang, Zhiyou Peng, Shoujun Tao, Jianliang Sun, Wenyuan Wang, Xuejiao Guo, Gonglu Liu, Xianzhe Luo, Yuan Chen, Yue Shen, Haixiang Ma, Peng Xu, Qinghua Li, Honghai Zhang, Zhiying Feng

**Affiliations:** ^1^Department of Anesthesiology, Women's Hospital, School of Medicine, Zhejiang University, Hangzhou, Zhejiang, China; ^2^Department of Pain Medicine, The First Affiliated Hospital, School of Medicine, Zhejiang University, Hangzhou, Zhejiang, China; ^3^Department of Anesthesiology, HangZhou First People's Hospital, School of Medicine, Zhejiang University, Hangzhou, Zhejiang, China; ^4^Department of Anesthesiology, Zhejiang Provincial People's Hospital, Hangzhou, Zhejiang, China; ^5^Department of Neurology, The Second Affiliated Hospital, School of Medicine, Zhejiang University, Hangzhou, Zhejiang, China; ^6^Department of Anesthesiology, Hangzhou First People's Hospital, Nanjing Medical University, HangZhou, Zhejiang, China; ^7^Department of Anesthesiology, The Fourth Clinical School of Medicine, Zhejiang Chinese Medical University, Hangzhou, China

## Abstract

Vesicular glutamate transporter type 2 (VGLUT2) is known to play an important role in mediating heat hyperalgesia induced by inflammation. However, the underlying mechanism for this activity is poorly understood. Cyclin-dependent kinase 5 (Cdk5), serving as a key regulator in modulating release of glutamate, acted a key player in the formation of heat hyperalgesia of inflammatory pain. However, it remains unknown whether there is a bridge between Cdk5 and VGLUT2 for mediating inflammatory pain. Therefore, we designed the experiment to determine whether VGLUT2 signaling pathway is involved in inflammatory pain mediated by Cdk5 in the inflammatory pain model induced by complete Freund's adjuvant (CFA). Our results showed that the coexpression of Cdk5/VGLUT2 in small- and medium-sized neuronal cells of the dorsal root ganglion (DRG) and spinal cord between days 1 and 3 following subcutaneous injection of CFA was significantly increased. Moreover, our study revealed that the expression of VGLUT2 protein in the DRG and spinal cord was remarkably increased between days 1 and 3 following CFA injection and was significantly reduced by roscovitine, a selective antagonist of Cdk5. Additionally, p25 but not p35, an activator of Cdk5, protein was significantly increased by CFA and reduced by roscovitine. Our findings suggested that VGLUT2/Cdk5 signaling pathway contributes to inflammatory pain mediated by Cdk5/p25.

## 1. Introduction

Pain caused by inflammation resulting from the peripheral or central nervous system remains a significant clinical problem and is often resistant to treatment with conventional analgesics [1–3]. It had been established that glutamate is the major excitatory neurotransmitter in the central nervous system and plays a key role in the processing of nociceptive pain [3]. Glutamate is transported into synaptic vessels by vesicular glutamate transporters (VGLUTs) composed of VGULT proteins 1–3 prior to its release from excitatory synapses [3]. Previous studies have established that VGLUT including subtype 1, 2, and 3 are expressed in distinct populations of glutamatergic neurons and play multiple roles in the central nervous system [3]. However, VGLUT2, but not VGLUT1 and 3, plays a key role in mediating pain hypersensitivity induced by inflammation and peripheral nerve injury [4–8]. The coexpression of VGLUT2 and two main nociceptors of calcitonin gene-related peptide (CGRP) and transient receptor potential channel (TRPV1) was observed in small- and medium-sized neurons of the dorsal root ganglion (DRG) in mice [4–8]. Additionally, VGLUT2 knock-out mice challenged with CFA demonstrates a complete loss of heat hyperalgesia, whereas mechanical hypersensitivity induced by CFA remains intact. Nevertheless, a detailed mechanism for VGLUT2 modulating the inflammation-induced heat hyperalgesia remains elusive [4].

Emerging evidence suggests that cyclin-dependent kinase 5 (Cdk5) and its activator p35, which can be split into p25 with more power than p35 to activate Cdk5 by calpain kinase in the central nervous system, play an important role in mediating inflammation-induced heat hyperalgesia [9–11]. The previous study demonstrated that Cdk5 can modulate CFA-induced heat hyperalgesia by controlling membrane trafficking of TRPV1 and phosphorylating vanilloid receptor 1 (VR1) in the dorsal root ganglion (DRG) of rats [4, 5]. The activities of Cdk5 and p35 in the spinal cord were significantly increased following peripheral injection of complete Freund's adjuvant (CFA). Furthermore, both Cdk5 kinase activity and heat hyperalgesia were inhibited by Cdk5/p35 knockdown or intrathecal administration of roscovitine [9–11]. Previous studies have suggested that presynaptic Cdk5 is the primary regulator of neurotransmitter release in the central nervous system [12].

Our previous studies revealed that increased levels of synaptophysin protein, an important presynaptic vesicle membrane protein that functions in release of neurotransmitters, are involved in mediating CFA-induced heat hyperalgesia mediated by Cdk5 in rats [13]. Furthermore, the recent study showed that VGLUT2-pH fluorescence colocalizes with synaptophysin at synaptic boutons and was involved in the trafficking of synaptic vesicles [14]. Altogether, this suggests that Cdk5 may mediate inflammation-induced heat hyperalgesia by controlling the release of neurotransmitters. Here, we report that the VGLUT2/Cdk5 signaling pathway contributes to the inflammatory pain by Cdk5.

## 2. Materials and Methods

### 2.1. Animals

All adult male Sprague Dawley rats (200–250 g) used in this study were obtained from the Animal Center of Nanjing Medical University (Nanjing, China). All experimental procedures were verified and approved by the Committee of Animal Use for Research and Education of Nanjing Medical University. Moreover, these procedures were performed in accordance with guidelines developed by the International Association for the Research on Pain [15]. Rats were placed in the room temperature of 22 ± 2°C and a standard 12/12 hours light/dark cycle, and food and water were available *ad libitum*. To minimize the animals we used in this research to avoid any unnecessary stress and pain, all animals were permitted to adapt to the housing facilities for 1 week prior to the experiments. Complete Freund's adjuvant CFA (100 *μ*L; Sigma, St. Louis, MO, USA) and saline (100 *μ*L) were injected into the plantar surface of the ipsilateral hind paws of rats, respectively (*n* = 6/group). The same volume of saline as used for the control group was injected into ipsilateral and contralateral paws of rats in the control group (*n* = 6/group).

### 2.2. Surgery and Drug Administration

Drugs were delivered in this study by spinal intrathecal injection as described by Yaksh and Rudy [16]. In brief, a catheter (PE-10 : 0.28 mm i.d. and 0.61 mm o.d.; Clay Adams, Parsippany, NJ, USA) was inserted at the lumbar level of the spinal cord between lumbar vertebrates 4 and 5 (L4 and L5) of the rats which were anesthetized before with 4% pentobarbital (40 mg/kg). A volume of 5 *μ*L of 2% lidocaine was delivered through the catheter for the recovery after anesthesia and surgery, followed by flushing with a volume of 15 *μ*L saline. Following this procedure, successful insertion of the catheter was further confirmed if the animal presented with impaired motor function of their hind legs at 10 s postlidocaine administrations. Following surgery, a volume of 5 *μ*L roscovitine (containing 100 *μ*g) (Sigma, St. Louis, MO, USA R7772) was dissolved in 10% DMSO and was delivered for 5 consecutive days to the rats in the experimental group, followed by rinsing of 15 *μ*L of sterile saline 0.5 h before single treatment with CFA (*n* = 6/group). The same concentration of roscovitine and DMSO was injected into the rats in the vehicle control group (*n* = 6) and the experimental group (*n* = 6).

### 2.3. Behavioral Test

Heat hyperalgesia was quantified by measuring paw withdrawal latencies (PWL) in response to radiant heat stimulation as previously described [13]. In brief, radiant heat was directed to the plantar surface of each hind paw through a 1 mm thick glass plate. In order to prevent tissue damage, a 20 second cutoff limit of duration was allotted for heat exposure. The time between the onset of the heat stimulus and a manifestation of paw withdrawal response was recorded as the thermal nociceptive latency period. Rats were allowed to adapt to the room for 30 min before the behavioral test. The thermal stimulus was then delivered to the ipsilateral and contralateral hind paws of rats in both groups after adaption. The time of PWL measurements was at 0 d, 6 h, 1 d, 3 d, and 5 d post-CFA or saline injection, which was recorded as the average result of 3 trials for each hind paw. The interval time between PWL measurements taken with the ipsilateral and contralateral paws was 5 minutes. Measurements of PWL in the DMSO and roscovitine-pretreatment groups were taken by the same method. The behavioral results showed that the heat hyperalgesia induced by CFA significantly decreased from 6 h to 5 d and reversed by roscovitine, which is in accordance with our previous studies [13].

### 2.4. Immunofluorescence

Rats from different groups were deeply anesthetized with sodium pentobarbital (>100 mg/kg i.p.) and were transcardially perfused with 10 ml PBS (pH 7.4), followed by 10 ml 4% paraformaldehyde. Tissues collected from ipsilateral segments L4–L6 of the DRG and spinal cord were immediately cryoprotected in 30% sucrose and sectioned transversely at 16 *μ*m thickness within a cryostat afterwards. The tissue sections were embedded with Tissue-Tek (Sakura Finetek, Torrance, CA, USA) and frozen in dry ice powder. The cross sections were sliced into 16 *μ*m thick sections at −28°C using a cryostat. Using double immunofluorescence for the examination, the sections were incubated overnight at 4°C with appropriate antibodies (anti-Cdk5 1 : 200, ab115812, and anti-VGLUT2, 1 : 200, ab79157; Abcam, Cambridge, MA, USA), followed by a second overnight incubation at 4°C with the corresponding secondary antibodies (1 : 200; Invitrogen, Carlsbad, CA, USA). An Axiovert/LSM 510 confocal scanning microscope (Carl Zeiss Microimaging, Inc., Germany) was used to examine the double-stained sections. After that, these sections were washed, and the fluorescence-labeled secondary antibodies (Goat anti-Mouse; goat anti-rabbit) were used for 1 h incubation at room temperature, respectively, and 9 slices from 3 different mice in each group. For each slice, Cdk5-positive and VGLUT2-positive (Cdk5(+)/VGLUT2(+)) cells from 4 randomly selected sections (250 *μ*m × 250 *μ*m) within DRG and spinal cord were manually counted [17].

### 2.5. Protein Extraction and Western Blot Analysis

DRG and spinal cord tissues from the L4–L6 ipsilateral sides of each treatment groups were collected, respectively, and immediately stored in ice-chilled lysis buffer (50 mM Tris, pH 7.4, 150 mM NaCL, 1.5 mM MgCL_2_, 10% glycerol, 1% Triton X-100, 5 mM EGTA, 0.5 *μ*g/ml leupeptin, 1 mM PMSF, 1 mM Na_3_VO_4_, 10 mM NAF, and a proteinase inhibitor cocktail). Homogenates were centrifuged at 12,000 rpm for 15 min at 4°C, and the BCA assay kit (Pierce, Rockland, IL, USA) was used to determine the protein concentrations. Each of the samples containing 30 *μ*g of protein were identified by sodium dodecyl sulfate polyacrylamide gel electrophoresis (SDS-PAGE) to determine the estimated molecular mass of the protein, and the gel was then transferred onto PVDF membranes. The membranes were then blocked with 1% bovine serum albumin (BSA) in TBST (50 mM Tris-HCL, pH 7.5; 150 mM NaCl, and 0.05% Tween 20) for 1 h at room temperature and incubated overnight at 4°C with the appropriate primary antibody (anti-VGLUT2, 1 : 500; Abcam, Cambridge, MA, USA, ab79157; anti-p35/p25, anti-p35, 1 : 300, Cell Signaling, MA, USA, 2680), second antibody for GAPDH and tubulin. Membranes were washed in TBST between each of the incubation. Blots were then incubated with 1 : 1000 dilution of horseradish peroxidase- (HRP-) conjugated secondary antibody (goat anti-rabbit or anti-mouse, respectively) for 1.5 h at room temperature. All bands were exposed to an X-ray film which was later digitally analyzed using NIH Image software, version 1.60. GADPH and tubulin proteins were used to normalize VGLUT2, p35, and p25 bands, respectively.

### 2.6. Statistical Analysis

All data are presented as mean ± SEM. SAS (version 8.01 software for Windows SAS Institute Inc., Cary, North Carolina, USA) was used to calculate statistical results. The time courses of effects were analyzed by two-way analysis of variance, followed by Dunnett's test. The VGLUT2 expression was analyzed using one-way analysis of variance, followed by the Student–Newman–Keuls' test, and the *P* value <0.05 was used to indicate statistical significance.

## 3. Results

### 3.1. Upregulated Expression of Cdk5/VGLUT2 in DRG Neurons

**Figure 1:**
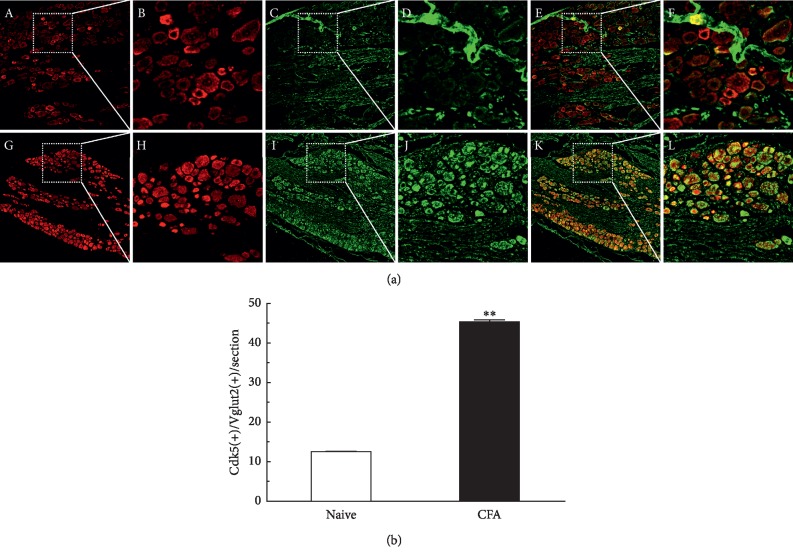
Coexpression of Cdk5 and VGLUT2 was increased in the DRG neuronal cells. Double-immunofluorescence staining for Cdk5 (red) and VGLUT2 (green). Compared with the control group by intraplantar injection of saline (A–F), the coexpression of Cdk5 and VGLUT2 was significantly increased in DRG neurons in the group on day 1 following intraplantar injection of CFA (G–L). Data shown represent mean ± SEM; ^*∗∗*^*P* < 0.01; *n* = 6/group.

To investigate the possible morphologic relationships between Cdk5 and VGLUT2, we examined the coexpression between Cdk5 and VGLUT2 from the DRG of the L4–L6 segments of the spinal cord. Compared with the rats in the control group by intraplantar injection of saline, the coexpression of Cdk5 and VGLUT2 was clearly elevated and mainly distributed in small- and medium-diameter neuron cells in the group on day 1 after intraplantar injection of CFA (Figure 1, ^*∗∗*^*P* < 0.01, *n* = 6/group). These results demonstrated that Cdk5 modulated inflammatory pain by closely interacting with VGLUT2.

### 3.2. Upregulated Expression of Cdk5/VGLUT2 in Spinal Cord Neurons

**Figure 2:**
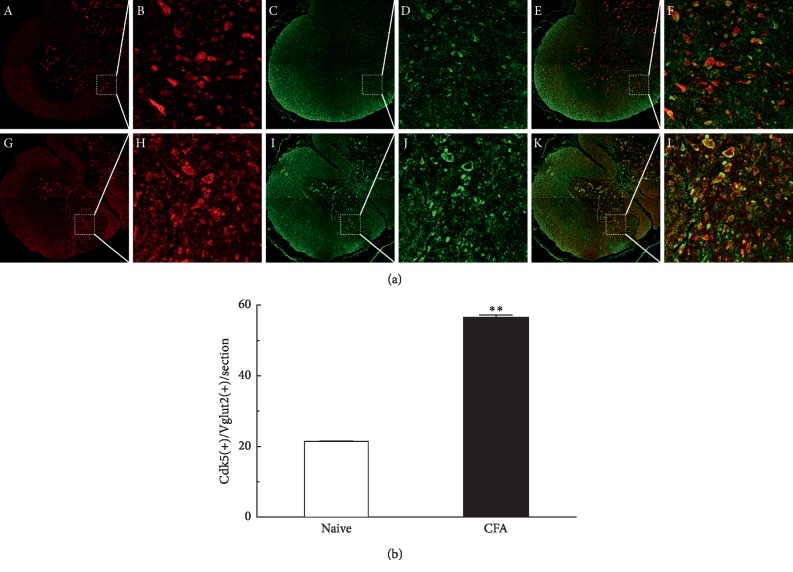
Coexpression of Cdk5 and VGLUT2 was increased in the spinal cord neuronal cells. Double-immunofluorescence staining for Cdk5 (red) and VGLUT2 (green). Compared with the control group by intraplantar injection of saline (A–F), the coexpression of Cdk5 and VGLUT2 was significantly increased in DRG neurons on day 1 following CFA injection (G–L). Data shown represent mean ± SEM; ^*∗∗*^*P* < 0.01; *n* = 6/group.

To further test the coexpression between Cdk5 and VGLUT2 in the spinal cord level, we examined and observed the coexpression of Cdk5 and VGLUT2 from the segments of the L4–L6 spinal cord using double-labeled immunofluorescence. The results showed that the coexpression of Cdk5/VGLUT2 was preferentially distributed in the gray matter area of spinal cord, which mainly transmitted the pain message from peripheral to central. Compared with the control group challenged with saline, the coexpression of Cdk5 and VGLUT2 was significantly increased in the rats of group on day 1 after intraplantar injection of CFA (Figure 2; ^*∗∗*^*P* < 0.01, *n* = 6/group).

### 3.3. Increased VGLUT2 Protein Expression in DRG Was Significantly Reduced by Roscovitine

**Figure 3:**
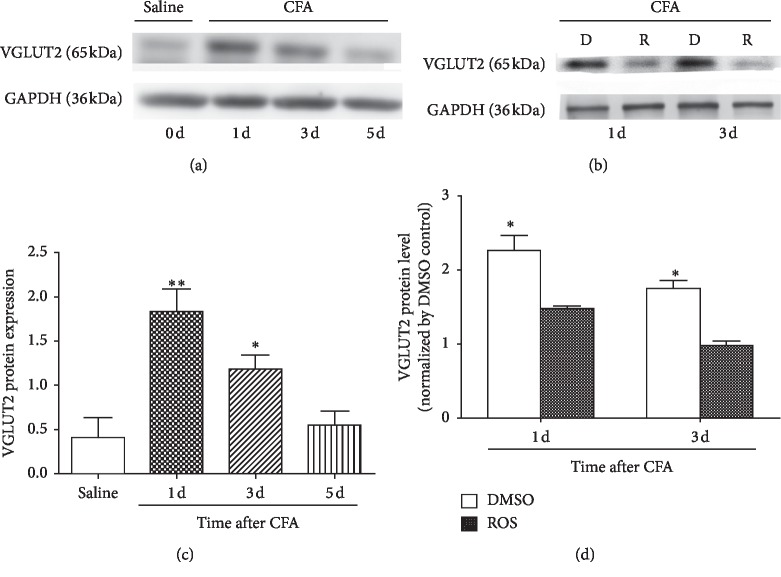
VGLUT2 protein expression was markedly increased in the DRG and reduced by roscovitine. Compared with the control group by intraplantar injection of saline, VGLUT2 expression was significantly increased from day 1 to day 3 following intraplantar injection of CFA. ^*∗*^*P* < 0.05 and ^*∗∗*^*P* < 0.01; *n* = 4/group A. Compared with the control group, by intrathecal injection of DMSO, the increased expression of VGLUT2 protein was significantly reduced by intrathecal injection of roscovitine between day 1 and day 3. Data shown represent mean ± SEM; ^*∗*^*P* < 0.05; *n* = 4/group B.

**Figure 4:**
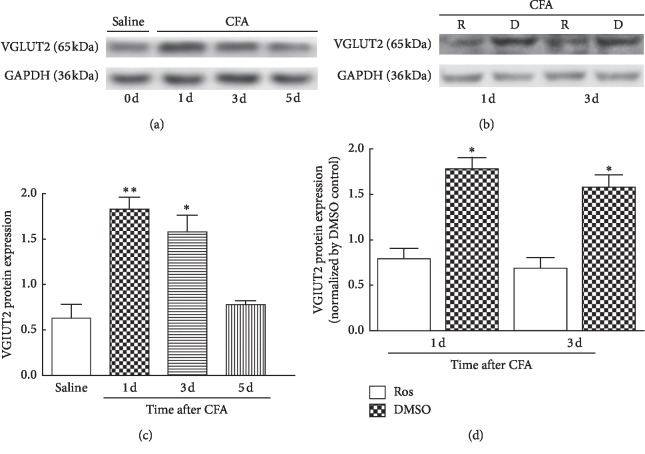
VGLUT2 protein expression was markedly increased in the spinal cord and reduced by roscovitine. Compared with the control group by intraplantar injection of saline, VGLUT2 expression was significantly increased from day 1 to day 3 after intraplantar injection of CFA. ^*∗*^*P* < 0.05 and ^*∗∗*^*P* < 0.01; *n* = 4/group A. Compared with the controls by intrathecal injection of DMSO, the increased expression of VGLUT2 protein was significantly reduced by intrathecal injection of roscovitine between day 1 and day 3 after intraplantar injection of CFA. Data shown represent mean ± SEM; ^*∗*^*P* < 0.05; *n* = 4/group B.

We next used Western blot analysis to investigate VGLUT2 protein expression in DRG neurons from the L4–L6 ipsilateral sides of spinal cord on days 0, 1, 3, and 5 after CFA injection. Our results showed that levels of VGLUT2 protein in DRG were significantly increased between days 1 and 3 after CFA injection (Figure 3, ^*∗*^*P* < 0.05, *n* = 4/group). Furthermore, the elevated levels of VGLUT2 protein between days 1 and 3 were obviously reduced by spinal intrathecal injection of roscovitine (Figure 4, ^*∗∗*^*P* < 0.01, *n* = 4/group).

### 3.4. Increased VGLUT2 Protein Expression in Spinal Cord Neurons Was Significantly Reduced by Roscovitine

In accordance with the results of VGLUT2 protein expression in DRG, the VGLUT2 protein from the L4–L6 ipsilateral spinal cord neurons challenged with CFA was significantly increased on days 1 and 3 compared with the control group. Moreover, the increased VGLUT2 protein was significantly reduced by spinal intrathecal injection of roscovitine (Figure 4,^*∗∗*^*P* < 0.01, *n* = 4/group).

### 3.5. p25, but Not p35, Protein Expression in Spinal Cord Neurons Was Significantly Increased by CFA

**Figure 5:**
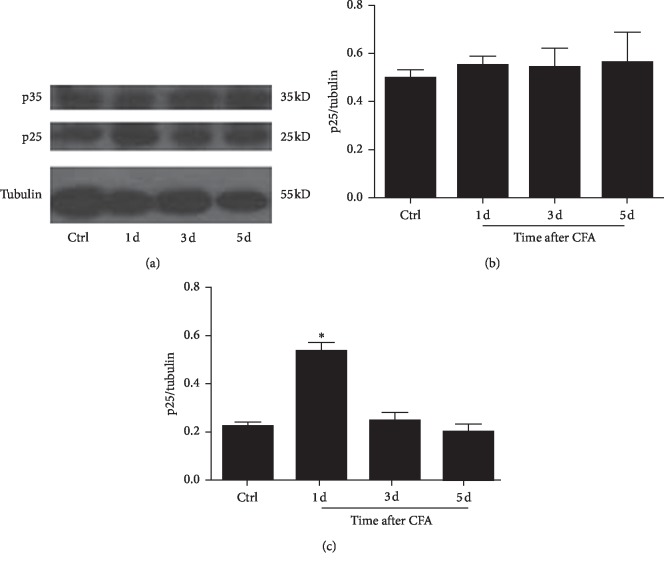
p25 but not p35 was markedly increased in the spinal cord. Compared with the control group treated with intraplantar injection of saline, p25 expression was significantly increased from day 1 after intraplantar injection of CFA. However, p35 expression was not significantly increased day 1 after intraplantar injection of CFA as compared with the control group treated with intraplantar injection of saline. Data shown represent mean ± SEM; ^*∗*^*P* < 0.05; *n* = 4/group.

To verify the role of p25 or p35 as an activator of Cdk5 in mediating the heat hyperalgesia by CFA, the p25 and p35 protein from the L4–L6 ipsilateral spinal cord neurons were examined, respectively. p25 protein from the L4–L6 ipsilateral spinal cord neurons challenged with CFA was significantly increased on days 1 and 3 compared with the control group. However, p35 remained markedly unchanged as compared with the control group (Figure 5, ^*∗∗*^*P* < 0.01, *n* = 4/group).

### 3.6. Increased p25 Protein Expression Was Significantly Reduced by Roscovitine

**Figure 6:**
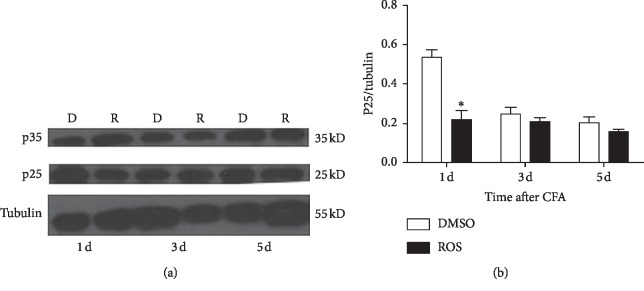
p25 not p35 was significantly reduced by roscovitine via p25 not p35. Compared with controls by intrathecal injection of DMSO in the group treated with intraplantar injection of CFA, the increased expression of p25 protein was significantly reduced by intrathecal injection of roscovitine in the group treated with intraplantar injection of CFA day 1. However, the administration of roscovitine produced no significant effects on p35 protein expression in the same group. Data shown represent mean ± SEM; ^*∗*^*P* < 0.05; *n* = 4/group B.

To explore the pathway inhibiting heat hyperalgesia by roscovitine, we, respectively, detected the protein changes of p25 and p35 after spinal intrathecal adminstration of roscovitine in the inflammation model induced by CFA. Basically, in agreement with the results of Figure 5, the expression of p25 was significantly reduced by roscovitine but not p35, suggesting that inhibition of heat hyperalgesia by Cdk5 inhibitor roscovitine was via inhibiting the activity of p25 and not p35 (Figure 6, ^*∗∗*^*P* < 0.01, *n* = 4/group).

## 4. Discussion

The present study preliminarily illustrated that the VGLUT2/Cdk5/P25 signaling pathway was involved in heat hyperalgesia induced by CFA in the DRG and spinal cord neurons. It is well established that glutamate is the major excitatory neurotransmitter in the CNS, and glutamate transmission plays a key role in nociceptive processing in inflammatory and neuropathological pain [1–3]. Glutamate storage in vesicles is controlled by VGLUTs, and glutamine, a precursor in the synthesis of L-glutamate, storing in small DRG neuron cells, accumulates 6 times larger than DRG neurons [18].

Some evidence demonstrated that Cdk5 acts a key role in mediating heat hyperalgesia induced by inflammation [9–11]. Moreover, previous results obtained by other investigators suggest that Cdk5 is a key regulator in mediation of neurotransmitters, including glutamate in the central nervous system [12]. Our published data demonstrated that synaptophysin, an important presynaptic vesicle membrane protein which mediates release of neurotransmitters, was involved in heat hyperalgesia mediated by Cdk5, suggesting that Cdk5 may modulate heat hyperalgesia induced by inflammation via controlling the release of neurotransmitters [13]. Another study further indicated that VGLUT2 was involved in the trafficking of synaptic vesicles by interacting with synaptophysin at synaptic boutons [14], which further showed that VGLUT2 may modulate the heat hyperalgesia by Cdk5 pathway. Thus, we examined the possible linkage between Cdk5 and VGLUT2 in modulating heat hyperalgesia induced by CFA using our current model.

In our research, the coexpression of Cdk5 and VGLUT2 was mainly distributed in small- and medium-sized neuronal cells of the DRG and spinal cord. It had been established that small- and medium-sized neuronal cells of the DRG and spinal cord are important mediators of inflammation and neuropathologic pain. Some studies showed that glutaminase (GLS) of the DRG, as an enzyme converting glutamine into glutamate for neurotransmission, can be significantly increased by innocuous stimuli in the inflammatory pain model induced by CFA [19]. Furthermore, another study suggested that deleting VGLUT2 of DGR led to the failure to the transmission of nociceptive pain [20].

Previous studies demonstrated that thresholds of heat hyperalgesia induced by CFA in mice with VGLUT2 knockout in the DRG were significantly increased compared to the thresholds in wild-type mice, suggesting that VGLUT2 plays a key role in mediating heat hyperalgesia induced by CFA [4]. In our study, both VGLUT2 and Cdk5 expression in the DRG and spinal cord neurons were significantly increased by CFA, and the increased VGLUT2 protein expressions were significantly reduced by roscovitine. Although the activities of Cdc2, Cdk2, and Cdk5 kinase are all inhibited by roscovitine [21], our previous findings suggested that in adult rats, roscovitine mainly affects the activity of Cdk5 and does not affect the activity of other Cdks in adult rats' neurons [13]. In addition, p25 but not p35 as the activator of Cdk5 contributed to CFA-induced heat hyperalgesia in our model. Taken together, our findings suggest that Cdk5 functions as the key regulator of VGLUT2 in mediating the heat hyperalgesia by CFA, and severing the linkage between Cdk5/p25 and the VGLUT2 signaling pathway may present a promising therapeutic strategy for diminishing inflammatory pain.

## Data Availability

The data used to support the findings of this study are available from the corresponding author upon request.

## Additional Points

Our current data failed to provide evidence to elucidate how Cdk5 mediates the neurotransmitters of glutamate to interact with VGLUT2 or how Cdk5 mediates the synaptic vesicles transportation by closely interacting with VGLUT2. In addition, we only observed the changes of heat hyperalgesia behavior and the protein expressions of both VGLUT2 and Cdk5/p35/25 by pharmacological intervention and molecular biology technology in our model to show the possible role of pain transmission mediated by VGLUT2, lacking the more specific method in our model to define the neural circuit of this pathway such as application of optogenetics technology [17]. Of course, we will design future experiments determining the mutual relationship between Cdk5 and VGLUT2 in mediating the inflammatory pain in our model by optogenetics technology.
